# Functional annotation of rare structural variation in the human brain

**DOI:** 10.1038/s41467-020-16736-1

**Published:** 2020-06-12

**Authors:** Lide Han, Xuefang Zhao, Mary Lauren Benton, Thaneer Perumal, Ryan L. Collins, Gabriel E. Hoffman, Jessica S. Johnson, Laura Sloofman, Harold Z. Wang, Matthew R. Stone, CommonMind Consortium, Schahram Akbarian, Jaroslav Bendl, Michael Breen, Kristen J. Brennand, Leanne Brown, Andrew Browne, Joseph D. Buxbaum, Alexander Charney, Andrew Chess, Lizette Couto, Greg Crawford, Olivia Devillers, Bernie Devlin, Amanda Dobbyn, Enrico Domenici, Michele Filosi, Elie Flatow, Nancy Francoeur, John Fullard, Sergio Espeso Gil, Kiran Girdhar, Attila Gulyás-Kovács, Raquel Gur, Chang-Gyu Hahn, Vahram Haroutunian, Mads Engel Hauberg, Laura Huckins, Rivky Jacobov, Yan Jiang, Jessica S. Johnson, Bibi Kassim, Yungil Kim, Lambertus Klei, Robin Kramer, Mario Lauria, Thomas Lehner, David A. Lewis, Barbara K. Lipska, Kelsey Montgomery, Royce Park, Chaggai Rosenbluh, Panos Roussos, Douglas M. Ruderfer, Geetha Senthil, Hardik R. Shah, Laura Sloofman, Lingyun Song, Eli Stahl, Patrick Sullivan, Roberto Visintainer, Jiebiao Wang, Ying-Chih Wang, Jennifer Wiseman, Eva Xia, Wen Zhang, Elizabeth Zharovsky, Kristen J. Brennand, Harrison Brand, Solveig K. Sieberts, Stefano Marenco, Mette A. Peters, Barbara K. Lipska, Panos Roussos, John A. Capra, Michael Talkowski, Douglas M. Ruderfer

**Affiliations:** 1 Division of Genetic Medicine, Department of Medicine, Vanderbilt University Medical Center, Nashville, TN USA; 2 Vanderbilt Genetics Institute, Vanderbilt University Medical Center, Nashville, TN USA; 3 Program in Medical and Population Genetics, Broad Institute of Harvard and Massachusetts Institute of Technology (M.I.T.), Cambridge, MA USA; 4 Center for Genomic Medicine, Massachusetts General Hospital, Boston, MA USA; 5 Department of Neurology, Massachusetts General Hospital and Harvard Medical School, Boston, MA USA; 6 Department of Biomedical Informatics, Vanderbilt University Medical Center, Nashville, TN USA; 7 Sage Bionetworks, Seattle, WA USA; 8 Division of Medical Sciences, Harvard Medical School, Boston, MA USA; 9 Pamela Sklar Division of Psychiatric Genomics, Department of Psychiatry, Icahn School of Medicine at Mount Sinai, New York, NY USA; 10 Icahn Institute for Data Science and Genomic Sciences, Department of Genetics and Genomic Sciences, Icahn School of Medicine at Mount Sinai, New York, NY USA; 11 Human Brain Collection Core, Intramural Research Program, NIMH, National Institutes of Health, Bethesda, MD USA; 12 Friedman Brain Institute, Icahn School of Medicine at Mount Sinai, New York, NY USA; 13 Psychiatry, JJ Peters VA Medical Center, Bronx, NY USA; 14 Department of Biological Sciences, Vanderbilt University, Nashville, TN USA; 15 Stanley Center for Psychiatric Research, Broad Institute of Harvard and M.I.T, Cambridge, MA USA; 16 Department of Psychiatry and Behavioral Sciences, Vanderbilt University Medical Center, Nashville, TN USA; 17 Division of Psychiatric Epigenomics, Department of Psychiatry, Icahn School of Medicine at Mount Sinai, New York, NY USA; 18 Seaver Autism for Research and Treatment, Department of Psychiatry, Icahn School of Medicine at Mount Sinai, New York, NY USA; 19 Department of Cell, Developmental and Regenerative Biology, Icahn School of Medicine at Mount Sinai, New York, NY USA; 20 Department of Neuroscience, Icahn School of Medicine at Mount Sinai, New York, NY USA; 21 Center for Genomic & Computational Biology, Duke University, Durham, NC USA; 22 Division of Medical Genetics, Department of Pediatrics, Duke University, Durham, NC USA; 23 Department of Psychiatry, University of Pittsburgh School of Medicine, Pittsburgh, PA USA; 24 Laboratory of Neurogenomic Biomarkers, Centre for Integrative Biology (CIBIO), University of Trento, Trento, Italy; 25 Neuropsychiatry Section, Department of Psychiatry, Perelman School of Medicine, University of Pennsylvania, Philadelphia, PA USA; 26 Neuropsychiatric Signaling Program, Department of Psychiatry, Perelman School of Medicine, University of Pennsylvania, Philadelphia, PA USA; 27 Department of Biomedicine, Aarhus University, Aarhus, Denmark; 28 Department of Mathematics, University of Trento, Trento, Italy; 29 National Institute of Mental Health, Bethesda, MD USA; 30 Department of Genetics, University of North Carolina at Chapel Hill, Chapel Hill, NC USA; 31 Department of Statistics and Data Science, Pittsburgh, PA USA

**Keywords:** Structural variation, Genomics, DNA sequencing, RNA sequencing, Neuroscience

## Abstract

Structural variants (SVs) contribute to many disorders, yet, functionally annotating them remains a major challenge. Here, we integrate SVs with RNA-sequencing from human post-mortem brains to quantify their dosage and regulatory effects. We show that genic and regulatory SVs exist at significantly lower frequencies than intergenic SVs. Functional impact of copy number variants (CNVs) stems from both the proportion of genic and regulatory content altered and loss-of-function intolerance of the gene. We train a linear model to predict expression effects of rare CNVs and use it to annotate regulatory disruption of CNVs from 14,891 independent genome-sequenced individuals. Pathogenic deletions implicated in neurodevelopmental disorders show significantly more extreme regulatory disruption scores and if rank ordered would be prioritized higher than using frequency or length alone. This work shows the deleteriousness of regulatory SVs, particularly those altering CTCF sites and provides a simple approach for functionally annotating the regulatory consequences of CNVs.

## Abstract (web summary)

Structural variants (SVs) contribute to the genetic architecture of many brain-related disorders. Here, the authors integrate SV calls from genome sequencing (n = 755) with RNA-seq data (n = 629) from post-mortem dorsal lateral prefrontal cortex to annotate the gene regulatory effects of SVs in the human brain and their potential to contribute to disease.

## Introduction

Structural variants (SVs) are a common and complex form of genetic variation that contribute substantially to phenotypic diversity and disease^1–3^. This contribution is notable in brain related disorders and traits such as schizophrenia, autism spectrum disorder (ASD), and cognition^4–8^. The advent of short-read genome sequencing has facilitated SV detection at nucleotide resolution and enabled a generation of large-scale reference studies^2,9,10^. Despite this progress, we still have a limited understanding of the functional impact of these variants, particularly for those seen infrequently in populations. Developing approaches to infer the functional consequences of SVs in the brain could have a profound impact on interpretation of genetic risk for complex brain disorders.

RNA-sequencing enables accurate measurement of transcription genome-wide^11^, and can thus facilitate a direct assessment of functional changes driven by genetic variants, including single nucleotide variants (SNVs) and SVs^12–15^. Previous work using earlier technologies has demonstrated that SVs have profound effects on expression with estimates of large copy number variants (CNVs) alone explaining 18% of variation in gene expression in cell lines^15^. Most work in this area has focused on common variants for which there is statistical power to identify direct association of a variant with expression of a gene^16^. However, recent analyses have suggested a substantial regulatory role for rare variants by identifying an enrichment of expression outliers in individuals harboring such variation^17,18^. In a small number of examples, rare SVs have shown the potential to alter the expression of genes both within and outside the SV locus with disease relevant phenotypic consequences. Such SVs often alter the regulatory landscape directly or through positional effects that change the three-dimensional structure of the genome. For example, the expression of *PLP1* is regulated by a downstream duplication and is associated with spastic paraplegia type 2 with axonal neuropathy^19^. To date, few samples have thus far been able to leverage both comprehensive SV detection from genome-sequencing and RNA-sequencing to explore the effects of rare SVs on expression genome-wide. Despite the importance of rare SVs in brain-related disorders and the tissue-specific nature of transcriptional regulation, efforts to understand the functional consequences of rare SVs in the brain have been impeded by the challenge in acquiring enough postmortem brain samples to be well-powered to quantify the dosage and regulatory effects of SVs.

The CommonMind Consortium (CMC; www.synapse.org/CMC) is a large collection of collaborating brain banks with over 1000 samples, including many with schizophrenia or bipolar disorder. Here, we leverage newly generated genome-sequencing data integrated with RNA-sequencing data from 629 samples that enable us to directly study the effects of rare SVs on expression in the brain. We show that SVs affecting regulatory elements are at significantly lower variant frequencies than expected, suggesting their potential to be deleterious. We also provide a quantitative characterization of the effects of SVs altering different regulatory elements have on expression. These results show that most complete gene deletions and duplications do not result in expression outliers and that genic intolerance to variation informs their functional impact. Finally, we build a model to infer the expression effects of SVs and use it to calculate a cumulative measure of regulatory disruption of an SV across all genes. When applied to a large independent SV reference data set^9,20^, the regulatory disruption score improves prioritization of pathogenic deletions beyond the common practice of considering frequency and SV length. Altogether, this work advances our understanding of the transcriptional consequences of SVs in the human brain and provides a framework for functionally annotating these variants to aid in disease studies.

## Results

### Evidence for selection against regulatory SVs

**Fig. 1:**
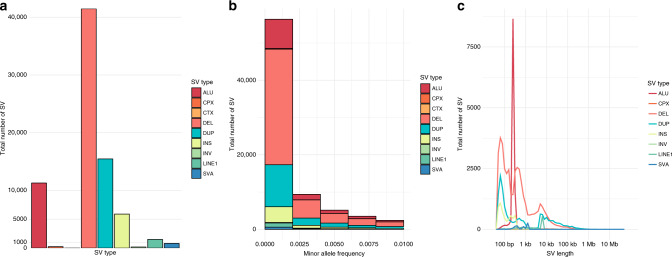
Details of CMC SV dataset. Characterization of high confidence rare (<0.5%) SV dataset stratified by **a** type of SV, **b** allele frequency, and **c** length (log10-scaled) colored by type of SV. SV types, include Alu (Alu), complex (CPX), translocation (CTX), deletion (DEL), duplication (DUP), insertion (INS), inversion (INV), long interspersed nuclear element-1 (LINE1), SINE-VNTR-Alu (SVA), including short interspersed nuclear elements, variable number tandem repeat, and Alu.

The SV detection pipeline identified 116,471 high-quality variants across 755 individuals. The final set of SVs predominantly consisted of CNVs (73%) and mobile element insertions (18%). The vast majority of SVs were small and rare (Fig. 1). The average length of SVs in this dataset was 7053 bp (median = 280 bp), with 78% of variants less than 1 kb. We next identified a subset of rare SVs (observed in <1% of individuals, AF < 0.5%, all SVs are treated as heterozygous), representing 88,819 variants. On average, individuals carried 338.4 rare SVs including 176.6 deletions and 69.3 duplications. These numbers differed by ancestry; individuals with African ancestries (mean = 615.9 SVs) carried substantially more rare SVs than individuals with European (mean = 190.5 SVs) or other ancestries, as expected.

**Fig. 2:**
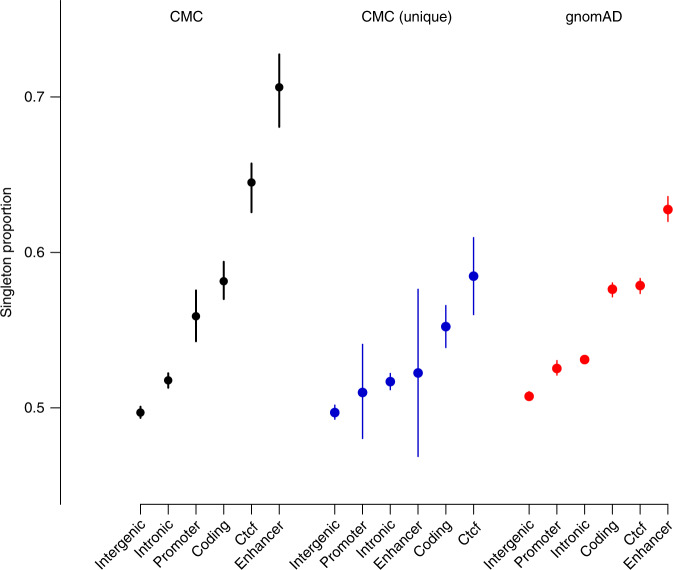
Genic and regulatory SVs occur at significantly lower frequencies. Proportion of variants that are seen only a single time with bootstrapped 95% confidence interval in the sample stratified by overlap with any annotation, allowing for multiple (CMC), only a single annotation (CMC unique) and any annotation in gnomAD SV.

We next sought to characterize how frequently SVs putatively alter gene dosage based on overlap with genes or regulatory elements. We defined a set of regulatory elements that included CTCF sites (*n* = 100,894), enhancers (*n* = 79,056) derived exclusively from brain tissue (see “Methods”) and promoters (2 kb upstream of the transcription start site [TSS]). Genes were defined as those in Ensembl v75 (*n* = 57,773) and where noted we split protein-coding genes (coding) from others which we label broadly as other transcribed products. For comparison, we defined two nonfunctional categories of SVs that did not overlap any annotation including those falling within introns (intronic) or those falling outside of any gene (intergenic). We note that these nonfunctional SV categories will include some proportion of SVs altering functional elements that were either not included, or that have not yet been identified, which should make our comparisons conservative. The allele frequency (AF) of SVs affecting protein-coding genes (AF = 0.00168, *p* = 7.42 × 10^−15^), enhancers (AF = 0.00123, *p* = 6.42 × 10^−30^), and CTCF sites (AF = 0.00161, *p* = 1 × 10^−16^) were significantly lower and singleton proportions were significantly higher than intergenic SVs (mean AF = 0.00193, Fig. 2) after matching on SV length to account for the known relationship between frequency and SV length (Supplementary Fig. 1, Supplementary Table 1, Wilcoxon test of AF distributions between the two annotation classes). These results were consistent across both deletions and duplications (Supplementary Table 1).

To explore the contributions of different functional elements to this result, we stratified SVs based on the specific annotations (e.g., coding and enhancer, Supplementary Fig. 2) to isolate those that alter combinations of annotations classes and those that uniquely alter a single annotation class (Supplementary Table 2). We identified a significant negative correlation between the total number of annotation classes affected and AF indicating that SVs with more potential to alter dosage are less likely to be tolerated (Supplementary Fig. 3). Further, we show that SVs exclusively affecting CTCF sites (AF = 0.00175, *p* = 1.48 × 10^−4^) when compared to intergenic variation showed comparable frequencies and significance to SVs that only affected protein-coding genes (AF = 0.00179, *p* = 1.48 × 10^−5^). These results are consistent across SV type and this difference in AF is seen when performing the same annotation of the gnomAD SV dataset of ~15 k samples called from genome-sequencing using the same pipeline^9^ (Fig. 2). These results suggest a strong selection against SVs that alter CTCF sites, consistent with previous work^21^.

### Transcriptional consequences of genic SVs

Among the samples with genome-sequencing, 629 individuals had RNA-sequencing data from the dorsal lateral pre-frontal cortex (DLPFC). RNA-sequencing was done across two cohorts (CMC and CMC_HBCC), results were consistent across cohorts as shown in many instances below. To quantify the transcriptional consequences of an SV, we defined expression in two ways. First, we calculated relative expression as the average expression of carriers divided by noncarriers. Second, we calculated *z*-scores using only noncarriers for calculating the mean and standard deviation to mitigate the effect of AF. We use both measures throughout, relying on relative expression in certain cases for interpretation but preferring *z*-scores for their statistical properties.

**Fig. 3:**
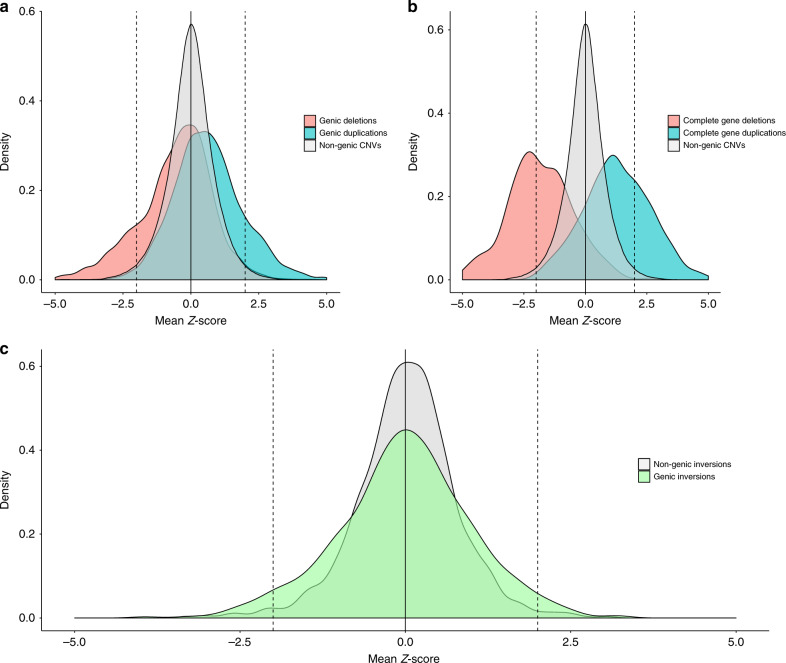
Genic SVs induce observable changes in expression. Expression presented as a *z*-score for **a** all CNV that overlap any proportion of the exonic sequence of a gene, **b** CNV that delete or duplicate 100% of the exonic sequence of a gene, and **c** all inversions with any gene overlap (green) compared to all other SVs (gray). Deletions are red, duplications are blue. The dashed lines are located at *z*-score of 2 and −2.

Previous literature has shown heterogeneity of effect on expression among putative loss-of-function (LoF) variants which is at least partly due to challenges in annotating functional impact^22,23^ Here, we expect complete deletions or duplications of all exons across all isoforms of a gene to result in an average 50% decrease or increase in expression, respectively. Relative expression calculated using read counts per million total reads (CPM) demonstrated the expected 50% decrease or increase from full gene deletions or duplications, on average (Supplementary Fig. 4). Deletions fit this expectation better than duplications, suggesting more variability among duplication calls and/or their functional effects. Normalization and linear covariate adjustment, which is necessary to account for confounders and batch effects (see “Methods”) alters the relative difference in expression among carriers to be closer to 25% while also reducing the variance, enabling clear demonstration of expression differences among individuals carrying full gene deletions or duplications (Supplementary Fig. 4). In general, expression was substantially lower across the full set of deletions and higher across the full set of duplications affecting genes (Fig. 3a), and these results were consistent across RNA-sequencing cohorts (Supplementary Fig. 5).

While there is an expectation for the expression effect of full gene deletions and duplications, the effects that other SVs may have on expression are not obvious. We identified a relationship between proportion of exonic sequence deleted/duplicated and expression (Supplementary Fig. 6) where the more exonic sequence deleted or duplicated the more extreme the expression difference. Of note, we saw more dramatic effects on expression for CNVs that altered the transcription start or 5′ end of the gene compared to transcription end or 3′ end regardless of proportion of exonic sequence affected (Supplementary Fig. 7). We defined expression outliers as those with *z*-scores greater than 2 or less than −2, and included any gene within 1 Mb of an SV assuming any affected base pair could regulate expression, similar to the standard window for identifying eQTLs. After Bonferroni correction for 60 tests (*p* < 0.00083), we identified significant excess of positive expression outliers for genic duplications (13.6%, *p* = 8 × 10^−181^, Fisher's exact test) and significant excess of negative expression outliers for genic deletions (14.2%, *p* = 3.1 × 10^−132^) when compared with CNVs of the same type but not affecting genes (Table 1). These results remained consistent whether we tested protein-coding genes or other transcribed gene products. Among the other SV classes with enough genic variants to be tested, only inversions showed a significant excess of expression outliers (Fig. 3c). This result was most significant when considering outliers in both directions (6.8%, *p* = 2.4 × 10^−5^, Table 1), with a larger contribution from positive expression outliers (4.1%, *p* = 4.5 × 10^−4^) than negative expression outliers (2.7%, *p* = 1.46 × 10^−2^). No effects were observed for insertions or Alu elements (Table 1).

**Table 1:** Genes affected by CNVs are significantly more likely to be expression outliers.

			*z*-Score > 2			*z*-Score < −2			|*z*-Score| > 2		
SV type	Annotation class	*N*	Outliers	Proportion	*p* Value	Outliers	Proportion	*p* Value	Outliers	Proportion	*p* Value
Deletions	Coding	1670	30	0.018	8.59E−02	237	0.142	3.1E-132*	267	0.160	3.62E−104*
	Intergenic	5,85,882	7980	0.014	9.99E−01	10,173	0.017	1.0E+00	18,153	0.031	1.00E+00
	Intronic	15,997	264	0.017	1.50E−03	286	0.018	5.1E−01	550	0.034	2.31E−02
	Other transcribed product	566	7	0.012	6.58E−01	114	0.201	1.5E−81*	121	0.214	2.28E−62*
Duplications	Coding	2374	324	0.136	7.99E−181*	36	0.015	7.9E−01	360	0.152	1.45E−122*
	Intergenic	2,30,007	3619	0.016	1.00E+00	3942	0.017	7.4E−02	7561	0.033	1.00E+00
	Intronic	4560	74	0.016	7.60E−01	76	0.017	6.0E−01	150	0.033	7.44E−01
	Other transcribed product	895	143	0.160	2.81E−89*	5	0.006	1.0E+00	148	0.165	7.45E−56*
Insertions	Coding	1395	21	0.015	2.57E−01	28	0.020	1.7E−01	49	0.035	1.16E−01
	Intergenic	82,414	1049	0.013	9.11E−01	1357	0.016	8.7E−01	2406	0.029	9.53E−01
	Intronic	829	12	0.014	3.75E−01	12	0.014	7.2E−01	24	0.029	5.57E−01
	Other transcribed product	337	7	0.021	1.45E−01	9	0.027	1.1E−01	16	0.047	4.24E−02
Inversions	Coding	515	14	0.027	1.46E−02	21	0.041	4.5E−04*	35	0.068	2.39E−05*
	Intergenic	2495	29	0.012	9.98E−01	42	0.017	9.8E−01	71	0.028	1.00E+00
	Intronic	57	0	0.000	1.00E+00	0	0.000	1.0E+00	0	0.000	1.00E+00
	Other transcribed product	162	5	0.031	9.02E−02	0	0.000	1.0E+00	5	0.031	6.63E−01
Alu	Coding	174	1	0.006	9.07E−01	6	0.034	6.8E−02	7	0.040	2.68E−01
	Intergenic	1,29,005	1751	0.014	3.69E−01	2116	0.016	7.9E−01	3867	0.030	5.94E−01
	Intronic	361	5	0.014	5.42E−01	4	0.011	8.4E−01	9	0.025	7.56E−01
	Other transcribed product	22	0	0.000	1.00E+00	1	0.045	3.1E−01	1	0.045	4.88E−01

Number and proportion of expression outliers by SV type and annotation. *p* Values are from Fisher’s Exact test (one-sided) comparing SVs in annotation class to others within SV type. * indicates significance after Bonferroni multiple test correction for 60 tests (*p* < 0.00083).

Our data showed an enrichment of expression outliers among genic SVs. However, we emphasize that the impact of structural rearrangement on expression is nonuniform and more complex than a simple accounting of the presence or absence of an SV. Even in the most extreme cases of 100% deletion or duplication, the affected protein-coding gene would only be considered an expression outlier for 61.7% of gene deletions and 34.1% of gene duplications (Fig. 3b). Furthermore, across all genic CNVs affecting protein-coding genes, only 16% of genic deletions and 15.2% of genic duplications result in gene expression outliers. These results demonstrate that the vast majority of known genic SVs would not be identified if restricted to expression outliers, so in contrast to this thresholding approach, a quantitative approach can more accurately assess the effects that SVs have on gene expression.

### Transcriptional consequences of regulatory SVs

Next, we quantified the transcriptional consequences of SVs that affect regulatory elements; this requires determination of a set of genes to analyze for each element. Our definition of promoters was necessarily gene-specific; however, for enhancers we explored numerous approaches concluding that genes predicted to be targets of enhancers from Hi–C^24^ was the most interpretable and useful for downstream analyses. Other approaches that we considered including the nearest gene, all genes within a shared topological associating domain (TAD) and all genes within a 1 Mb window showed similar results. We therefore included 90,015 enhancer-gene pairs covering 6535 genes and 32,803 enhancers predicted from PsychENCODE Hi–C data^24^. To capture the relative contributions of all annotations, we tested the relationship between gene expression *z*-scores and SV annotations with a joint linear model that included proportion of exonic sequence, promoter proportion, sum proportion of all affected enhancers, whether SV and gene were within the same TAD and SV length. The most significant contributor to expression was the proportion of the exonic sequence affected (deletions: beta = −1.78, *p* = 9.9 × 10^−158^; duplications: beta = 0.78, *p* = 3 × 10^−109^). Expression was significantly and positively correlated with the proportion of a promoter that was affected by CNVs with deletions leading to lower expression (beta = −0.17, *p* = 3.4 × 10^−3^) and duplications leading to higher expression (beta = 0.37, *p* = 2.5 × 10^−30^). Further, expression was significantly correlated with the cumulative sum of enhancer sequence that was affected by an SV only in duplications, but both deletions and duplications led to decreased expression (deletions: beta = −0.02, *p* = 0.067; duplications: beta = −0.02, *p* = 8.1 × 10^−9^). The presence of the SV and the gene within the same TAD contributed significantly and directionally to expression in deletions (beta = −0.009, *p* = 5.7 × 10^−5^) but not duplications (beta = 0.005, *p* = 0.21). The effects of these variables on expression were consistent across cohort (Table 2) and while proportion of exonic sequence provided the strongest contributor, the effects of *cis*-regulatory elements remained significant in duplications and to a lesser extent in deletions after excluding all genic SVs (Supplementary Table 3).

**Table 2:** Genic and regulatory features significantly contribute to predicting transcriptional consequences of CNVs.

						*CMC*				*CMC_HBCC*			
CNV class	Variable	Beta	SE	T	P	Beta	SE	T	P	Beta	SE	T	P
Deletions	Exonic Proportion	−1.7762	0.0664	−26.77	9.9E−158	−2.0563	0.0852	−24.12	1.8E−128	−1.5571	0.0939	−16.59	8.68E−62
	Enhancer sum	−0.0152	0.0083	−1.83	6.7E−02	−0.0093	0.0094	−0.99	3.2E−01	−0.0188	0.0112	−1.69	9.14E−02
	Promoter proportion	−0.1726	0.0589	−2.93	3.4E−03	−0.1719	0.0747	−2.30	2.1E−02	−0.0872	0.0843	−1.04	3.01E−01
	SV Length	−2.14E−07	1.46E-08	−14.70	6.9E−49	−1.92E−07	1.70E−08	−11.26	2.0E−29	−1.89E−07	3.42E−08	−5.53	3.19E−08
	Within TAD	−0.0090	0.0022	−4.03	5.7E−05	−0.0113	0.0031	−3.61	3.0E−04	−0.0046	0.0028	−1.65	9.83E−02
Duplications	Exonic Proportion	0.7825	0.0352	22.22	3.0E−109	1.1285	0.0546	20.67	1.0E−94	0.5043	0.0442	11.42	3.43E−30
	Enhancer sum	−0.0157	0.0027	−5.77	8.1E−09	0.0015	0.0062	0.24	8.1E−01	−0.0164	0.0030	−5.42	5.84E−08
	Promoter proportion	0.3735	0.0326	11.45	2.5E−30	0.3523	0.0509	6.92	4.5E−12	0.3438	0.0403	8.54	1.37E−17
	SV Length	3.99E-07	2.60E-08	15.34	4.6E−53	4.07E−07	3.52E−08	11.58	5.3E−31	3.05E−07	3.68E−08	8.30	1.04E−16
	Within TAD	0.0046	0.0036	1.26	2.1E−01	0.0072	0.0052	1.38	1.7E−01	0.0038	0.0045	0.86	3.89E−01

Coefficients of linear regression model to predict expression *z*-scores in deletions and duplications, across all samples and stratified by cohort.

### Integrating transcriptional consequences and gene intolerance

**Fig. 4:**
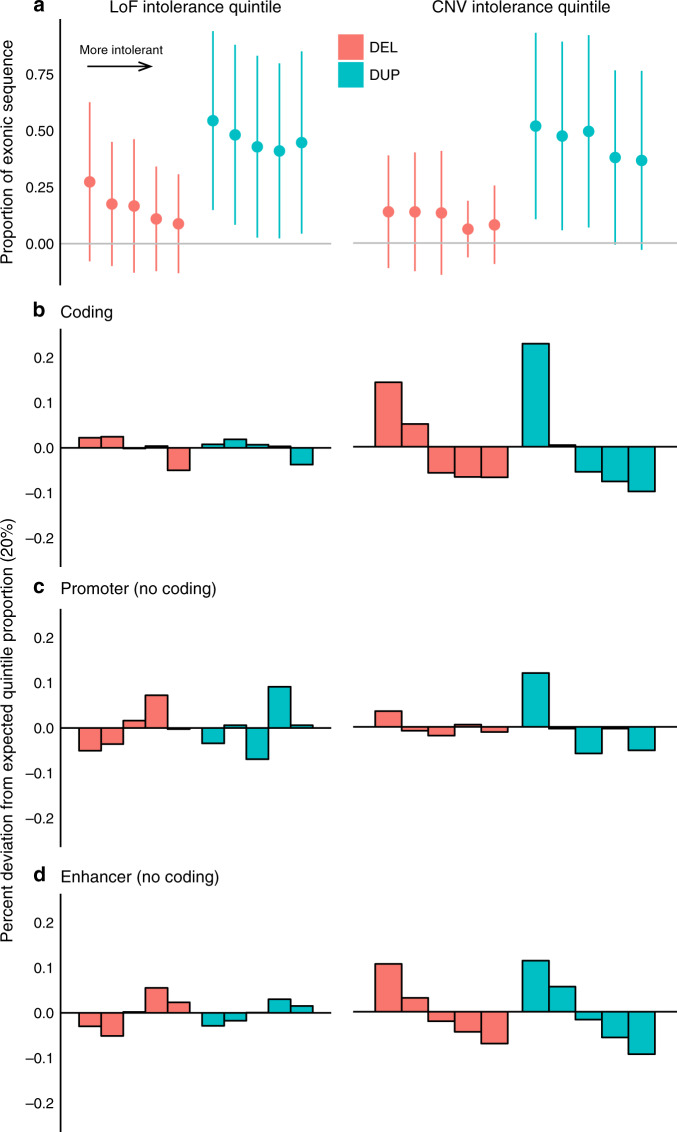
Genes intolerant to variation are less likely to be affected by genic or regulatory SVs. Each plot stratifies genes using either the LoF intolerance metric or the CNV intolerance metric that have been split into quintiles (20% bins) ordered left to right from least to most intolerant genes and by deletion (red) and duplication (blue). The plots show the effect of this stratification on **a** the proportion of the exonic sequence that is affected showing mean and standard deviation, **b** the deviation from the expected 20% of CNV that alter exonic sequence, **c** the deviation from expected for noncoding CNV that alter promoters, and **d** the deviation from expected for noncoding CNV that alter enhancers.

To better understand the relationship between our variant annotations in the context of the genes affected, we incorporated two distinct measures of genic intolerance to variation: (1) gene intolerance to CNVs defined empirically from exome-sequencing in nearly 60,000 individuals^25^, and (2) a measure of gene intolerance to LoF variation generated from a sample of ~141,000 individuals^20^. Several significant relationships between the functional effects of SVs and the intolerance of the genes affected existed. SVs that disrupted intolerant genes were significantly more likely to alter a smaller proportion of the exonic sequence (pLoF = 2.42 × 10^−38^, pCNV = 1.31 × 10^−33^, Spearman correlation test of intolerance and proportion of exonic sequence affected, Fig. 4a). Intolerant genes were also significantly less likely to have a genic SV (pLoF = 2.4 × 10^−38^, pCNV = 9.36 × 10^−34^, Wilcoxon test of gene metric by whether SV affects exonic sequence or not, Fig. 4b). Consistent with previous literature showing intolerance to dosage changes in either direction^25^ we saw intolerant genes less likely to be affected by both deletions (pLoF = 7.41 × 10^−29^, pCNV = 3.02 × 10^−26^) and duplications (pLoF = 3.81 × 10^−22^, pCNV = 1.57 × 10^−46^). Further, when restricting to SVs that only alter regulatory elements and not exonic sequence, we identified a significant decrease in the number of enhancers affected by SVs in genes with higher intolerance, although this was only observed for the CNV intolerance metric (pLoF = 0.62, pCNV = 7.23 × 10^−22^, Fig. 4d). We did not find any effects from promoter SVs in either metric (pLoF = 0.35, pCNV = 0.37, Fig. 4c). Combined with the differences seen by CNV type these results may indicate unique properties of these metrics and what they reflect (e.g., haploinsufficiency vs. dosage sensitivity). In general, as with single nucleotide variation, genic measures of intolerance should help functionally annotate SVs.

### A model to annotate SVs from predicted dosage and gene intolerance

**Fig. 5:**
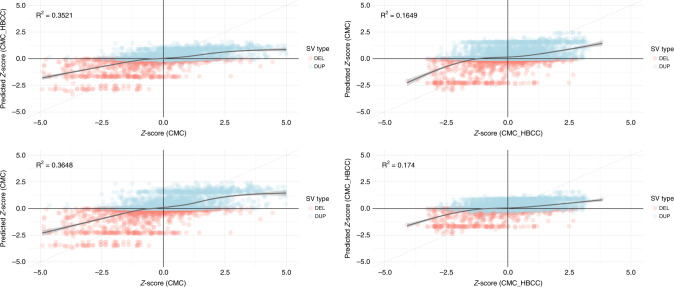
Transcriptional consequences of rare CNVs can be significantly predicted. SV expression prediction performance and associated *R*^2^ from building the same linear model using different training and test datasets. **a** CMC into CMC_HBCC, **b** CMC_HBCC into CMC, **c** CMC into CMC, and **d** CMC_HBCC into CMC_HBCC. The best fit line with confidence interval was produced using generalized additive model smoothing.

Having demonstrated a significant role for SVs in altering expression, we sought to test whether this model could be used to predict expression effects of SVs in independent samples. We split our DLPFC sample by cohort (CMC and CMC_HBCC, see “Methods”) and constructed the linear model described previously in each subset and then applied that model to SVs in the other set to infer expression effects. We identified significant correlation between the true expression value and the predicted value across all four pairwise comparisons (*R*^2^_CMC_HBCC→CMC_ = 0.35, *R*^2^_CMC→CMC_HBCC_ = 0.17, *R*^2^_CMC→CMC_ = 0.36, *R*^2^_CMC_HBCC→CMC_HBCC_ = 0.17, Fig. 5) with deletions (particularly when tested in CMC) consistently performing better.

**Fig. 6:**
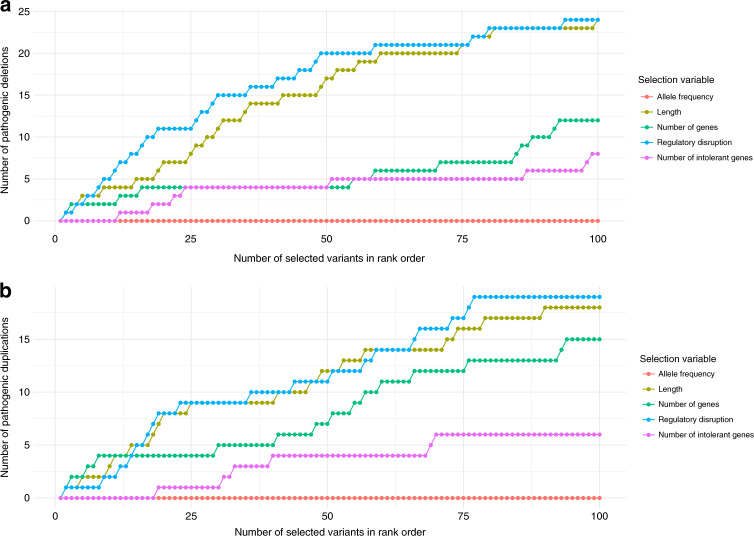
Regulatory disruption scores prioritize pathogenic CNVs better than standard annotations. Number of pathogenic variants defined as 50% overlap with known pathogenic variant in ClinGen (84 deletions and 84 duplications) identified based on rank ordering deletions **a** and duplications **b** by length (yellow), number of genes deleted (green), number of intolerant genes deleted (purple) allele frequency (red), and regulatory disruption (blue). Where multiple variants had the same value, the order was random.

Leveraging this model and the previously used measure of genic intolerance to LoF variation, we built an aggregate regulatory disruption score that was the sum of the predicted expression *z*-scores for each gene weighted by the gene’s intolerance metric (normalized between 0 and 1 with 1 being most intolerant) to annotate SVs. We then applied our model to annotate 210,244 variants in the gnomAD SV dataset^9^ after restricting to CNVs that were below 1% frequency. Of those, 31,492 (15%) were predicted to alter the expression of at least one protein-coding gene where we had an intolerance metric, 20,236 of these variants were deletions and 11,256 were duplications. We considered a deletion or duplication in gnomAD as pathogenic if it overlapped at least 50% of a CNV of the same type (3454 deletions and 1894 duplications) labeled pathogenic for neurodevelopmental disorders (developmental delay, intellectual disability, or autism) in ClinGen (downloaded from UCSC Genome Browser June 2019). There were 84 deletions and 84 duplications that met this criterion (39 deletions and 33 duplications overlapped 100% of the pathogenic ClinGen variant, as gnomAD includes some individuals with neuropsychiatric disorders). This set of pathogenic CNVs had significantly larger regulatory disruption scores in the direction of the dosage change with deletions having a more severe reduction in expression among intolerant genes due to these deletions (*p* = 1.78 × 10^−26^, mean score in pathogenic deletions = −5.21, mean score in nonpathogenic deletions = −0.18, Wilcoxon test) and duplications having a dramatic increase (*p* = 9.76 × 10^−39^, mean score in pathogenic duplications = 12.67, mean score in nonpathogenic duplications = 0.52). Despite ascertainment bias leading to longer CNVs being more likely to overlap pathogenic variants, prioritizing variants by regulatory disruption would identify more pathogenic deletions than prioritizing by length, with four of the top ten variants being pathogenic if ranked by length (two complete overlaps) and seven by regulatory disruption (five complete overlaps). The regulatory disruption score also better prioritized pathogenic deletions than number of all genes affected, number of intolerant genes (top 10%) affected and AF, which has limited utility since most deletions (53% or 10,776) have the same frequency, as singletons. (Fig. 6a, Supplementary Data 1). For duplications, the regulatory disruption score performs similarly to length but still outperforms other measures (Fig. 6b, Supplementary Data 2). These results indicate the potential of this metric to contribute to improved prioritization of disease causing CNVs, particularly deletions.

## Discussion

The integration of genome and transcriptome data on postmortem brains from the CMC has provided one of the first opportunities for large-scale characterization of the impact of rare SVs on expression in the brain. Here, we demonstrate evidence of selection on rare regulatory SVs, particularly those that alter CTCF binding sites. We found a clear and predictable role for genic and regulatory SVs in altering expression, and we showed that the degree of expression influence is shaped by the intolerance of a gene to deleterious variation. These results suggest the potential to functionally predict and annotate the consequences of SVs on expression. Illustrating this potential, we derived a model to infer expression effects of SVs in independent samples, and applied it to the largest SV resource currently available. This provided evidence that annotating SVs by their regulatory burden could aid in prioritizing disease relevant variants.

Selection maintains deleterious variation at lower allele frequencies, enabling the use of frequency as a proxy for implicating variant classes that may contribute to phenotypes negatively affecting fitness. Here, we showed that SVs overlapping brain regulatory elements including enhancers and CTCF sites were seen at significantly lower frequencies than SVs that were intronic or intergenic. This result remained true even after accounting for SV length and could also be shown in an independent and substantially larger dataset (gnomAD). Regulatory variants, particularly SVs, have the potential to alter the expression of many genes and previous work has already implicated de novo variants in fetal enhancers in risk for neurodevelopmental disorders^26^. As an example, a deletion of a CTCF site could enable enhancer activity of nearby genes, a mechanism known as enhancer hijacking that is particularly common in cancer^27^. Based on comparison of frequency and singleton proportion within our sample and replicated within gnomAD, it appears that SVs altering brain relevant CTCF sites are as infrequent as genic SVs; this suggests that a substantial proportion may be deleterious and being actively removed from the population by selection. Previous work has directly tested this hypothesis using very different data and approaches and found similar results^21^. With the growing amount of functional and genomic data, assessing the role of SVs on regulatory elements, particularly CTCF sites, in disease will further assess the validity and importance of this finding.

Rare SVs show substantial but variable effects on expression that can be quantified. While identifying potential carriers of rare functional SVs using expression outliers is a practical and valid approach, in our data using this definition would result in the identification of only a small proportion of all genic SVs (including missing most full gene CNVs) and a substantially smaller proportion of regulatory SVs. The approach taken here requires genomic annotations to implicate regulatory effects and an assumption that the annotated elements are functional. Despite these limitations, it is clear that both genic and regulatory SVs have significant functional impact that can be quantified and used to infer expression effects of SVs in independent datasets. These effects are strongest when altering exonic sequence, but are significant when altering only promoter and enhancer sequence as well. In all cases the effect is proportional to the amount of functional sequence affected.

We specifically required that regulatory element annotation be gene-specific to facilitate prediction of enhancer-gene associations. In other words, we showed that we can quantify the effect of a CNV on a specific gene. As ongoing efforts to understand the gene-specific functions of enhancers improve regulatory annotations, so too will the approaches in this paper be improved in accuracy and expanded beyond the roughly 30% of genes we were able to include. Better enhancer-gene target annotations would increase the number of CNVs that could be predicted and the performance of those predictions. We identified a significant negative effect of duplications altering enhancers. This result presents a potentially intriguing implication regarding the direction of regulatory effect of enhancer duplications. One potential concern is that this effect is seen substantially more strongly in one of our two cohorts suggesting a heterogenous or batch effect. Further work is required to better understand the role of enhancer duplications on expression. We did not include CTCF sites in the prediction model as it was not clear how to directly link them to individual genes, however, based on the likely deleteriousness of CTCF SVs, we anticipate effects on potentially many genes and quantifying those effects is an area of future work. We did include TAD annotations as a surrogate for potential contribution to the expression consequence of an SV, and we identified a significant magnification in effect on expression when a deletion and gene were within the same TAD, pointing to the importance of TADs in the regulatory landscape. We present a simple linear model that can meaningfully predict expression effects of rare SVs and anticipate that improvements in regulatory annotation and more sophisticated modeling will further the ability to make these predictions.

Improvements in annotation have enabled better prioritization of variants that may cause or increase risk of disease. One avenue to improved annotation has been leveraging large numbers of sequenced individuals in order to quantify the intolerance of each gene to deleterious variation. Here, we show that gene level intolerance metrics also inform regulatory effects of SVs. Not surprisingly, SVs are less likely to affect intolerant genes but when they are the proportion of the exonic sequence affected is significantly smaller. Combining our SV expression predictions and previously generated gene intolerance measures allowed us to annotate the overall regulatory disruption of an SV by weighting the predicted expression consequences by the relative deleteriousness of the gene affected. For example, a complete deletion of a gene where LoF variants are rarely or never seen will be weighted higher than that of a gene that is frequently knocked out in the population. To demonstrate the potential utility of this regulatory disruption score, we annotated all CNVs in the gnomAD SV dataset and showed that our annotations were correlated with variants that substantially overlapped those that have previously been classified as pathogenic. Further, rank ordering variants by the most extreme regulatory disruption scores enriched for pathogenic variants that would have not been identified by length, frequency or number of genes. These variants would also not have been identified simply by looking for CNVs that affected intolerant genes as the majority of both the deletions (57/84) and duplications (46/84) were not among the 1.6% of CNVs in gnomAD SV that affected at least one of the 10% most intolerant genes. We note that the regulatory disruption score performs better for deletions than for duplications which could be a product of our improved prediction accuracy and/or related to the greater challenges in assigning pathogenicity to duplications. At present, the regulatory disruption score provides an additional metric that may serve to highlight potential pathogenic CNVs not highlighted by other approaches. These variants would still require the same clinical scrutiny applied to any other prioritized CNVs for determining true pathogenicity. Therefore, potential exists for annotation of regulatory disruption to improve prioritization of disease causing or risk increasing SVs; however, further work with clinical or disease samples will be required to fully assess the added value of this approach.

Our work has several limitations, including an assumption that effects of SVs on expression are largely products of proportional alterations on genic and regulatory genomic content. Our predictions demonstrate that this assumption holds on average; however, we anticipate gene and regulatory element specific effects to exist. This assumption was necessary because, in the case of rare SVs, estimating individual variant effects is underpowered. Further, it is likely that the proportional relationship between exonic sequence and expression effect is at least in part due to the use of constituent gene products and future use of isoform level expression should provide more specificity of SV effect. We also leverage assumptions about the direction of effect for large CNVs, and show that this direction of effect extends to smaller variants. We largely focus on CNVs given the expected directional effects as well as the predominance of these variants among our confident calls, which better powers these analyses. We can show expression effects of inversions; however, these effects are substantially smaller and not in a specific direction. We also see very similar patterns of deleteriousness, through lower frequencies, in other classes of SVs that alter regulatory elements and genes. SVs that are not CNVs are still a challenge to call accurately and harder to validate given the smaller set of previously identified variants of high confidence. We anticipate improvements in calling these variants to lead to the ability to better define their functional role in the near future.

This effort was entirely focused on the brain: the expression data were from brain tissue and all of the regulatory elements included were identified in the brain. Some findings, such as the deleteriousness of CTCF sites and the basic prediction model, likely hold true across other tissues as the assumptions are not tissue-specific and CTCF sites are relatively shared across tissues. For other results, such as the expression consequences of enhancer SVs or the regulatory disruption scores, it is unclear how well they will generalize given the tissue-specific nature of enhancers and the fact that brain expressed genes are among the most conserved and intolerant to variation. It is, therefore, advisable to develop these models in a tissue-specific manner wherever possible.

In conclusion, genome-sequencing and RNA-sequencing when combined in the same samples can be used to interpret the transcriptional consequences of SVs for improved annotation of a variant class that, despite its clear importance, remains difficult to quantify its functional effect.

## Methods

### Cohorts

Samples were included from two different cohorts. The CMC study is a combined collection of brain tissues from the Mount Sinai NIH Brain Bank and Tissue Repository (*n* = 127), The University of Pennsylvania Brain Bank of Psychiatric Illnesses and Alzheimer’s Disease Core Center (*n* = 62) and The University of Pittsburgh NIH NeuroBioBank Brain and Tissue Repository (*n* = 139). Tissue for the collection was dissected at each brain bank and shipped to the Icahn School of Medicine at Mount Sinai (ISMMS) for nucleotide isolation and data generation in one facility in order to reduce site-specific sources of technical variation. Postmortem tissue from schizophrenia and bipolar disorder cases were included if they met the diagnostic criteria in DSM-IV for schizophrenia or schizoaffective disorder, or for bipolar disorder, as determined in consensus conferences after review of medical records, direct clinical assessments, and interviews of care providers. Cases that had a history Alzheimer’s disease, and/or Parkinson’s disease, or acute neurological insults (anoxia, strokes, and/or traumatic brain injury) immediately prior to death, or were on ventilators near the time of death, were excluded. The CMC_HBCC study includes brain samples from the NIMH Human Brain Collection Core (*n* = 445). All specimens were characterized neuropathologically, clinically and toxicologically. A clinical diagnosis was obtained through family interviews and review of medical records by two psychiatrists based on DSM-IV criteria. Nonpsychiatric controls were defined as having no history of a psychiatric condition or substance use disorder. Among the 773 samples used here, there are 505 males and 268 females. Self-reported ancestries consisted of 484 European, 264 African, 15 Hispanic, 9 Asian and 1 other. Forty-eight percent of the samples had a psychiatric diagnosis (287 schizophrenia, 83 bipolar disorder) and the remaining 403 were considered controls^28^. All research complied with ethical regulations and was approved by the Vanderbilt University Medical Center Institutional Review Board (IRB# 161488).

### DNA Isolation

DNA for all 773 samples was isolated from approximately 50 mg dry homogenized tissue from the dorsal lateral prefrontal cortex (DLPFC). All tissue samples had corresponding tissue samples that were isolated for RNAseq. All DNA isolation was done using the Qiagen DNeasy Blood and Tissue Kit (Cat#69506) according to manufacturer’s protocol. DNA yield and genomic quality number (GQN) was quantified using Thermo Scientific’s NanoDrop and the Fragment Analyzer Automated CE System (Advanced Analytical). Totally, 96 samples had a GQN < 4, but were not excluded from genome-sequencing. The mean yield was 9.9 µg (SD = 10.4) and the mean GQN was 5.6 (SD = 1.47).

### DNA library preparation

All samples were submitted to the New York Genome Center for genome-sequencing, where they were prepared for sequencing in randomized batches of 95. The sequencing libraries were prepared using the Illumina PCR-free DNA sample preparation Kit. The insert size and DNA concentration of the sequencing library was determined on Fragment Analyzer Automated CE System (Advanced Analytical) and Quant-iT PicoGreen (ThermoFisher) respectively. A quantitative PCR assay (KAPA), with primers specific to the adapter sequence, was used to determine the yield and efficiency of the adapter ligation process.

### Genome-sequencing library preparation and sequencing

Libraries for genome sequencing were generated from 100 ng of genomic DNA using the Illumina TruSeq Nano DNA HT sample preparation kit. Genomic DNA were sheared using the Covaris sonicator (adaptive focused acoustics), followed by end-repair, bead-based size selection, A-tailing, barcoded-adapter ligation followed by PCR amplification. Final libraries were evaluated using qPCR, picogreen and Fragment analyzer. Libraries were sequenced on a 2 × 150 bp run of a HiSeq X instrument.

### Genome-sequencing pipeline

Paired-end 150 bp reads were aligned to the GRCh37 human reference using the Burrows–Wheeler Aligner (BWA-MEM v0.78) and processed using the best-practices pipeline that includes marking of duplicate reads by the use of Picard tools (v1.83, http://picard.sourceforge.net), realignment around indels, and base recalibration via Genome Analysis Toolkit (GATK v3.2.2). Variants were called using GATK HaplotypeCaller, which generates a single-sample GVCF. To improve variant call accuracy, multiple single-sample GVCF files were jointly genotyped using GATK GenotypeGVCFs, which generated a multi-sample VCF. Variant Quality Score Recalibration (VQSR) was performed on the multi-sample VCF, which added quality metrics to each variant that can be used in downstream variant filtering.

### SV discovery

SVs were detected using a discovery pipeline^29^ that relies upon an ensemble of SV detection algorithms to maximize sensitivity, followed by a series of filtering modules to control the overall false-discovery rate (FDR) and refine variant predictions. In brief:

1. Raw SV calls collection: Five algorithms that used discordant pair-end reads (PE) and split reads (SR) to predict SVs, i.e., DELLY^30^ (v0.7.5), LUMPY^31^ (v0.2.13), Manta^32^ (v1.01), Wham^33^ (v1.7.0) and MELT^34^ (v2.1.4), were executed in per-sample mode with their default parameter. A series of read depth-based (RD) algorithms were also applied for CNV detection, including CNVnator^35^ (v0.3.2), GenomeSTRiP^36^ (v2), and a custom version of cn.MOPS^29^. These algorithms were applied to male and female samples separately, each in ~100-sample batches. For each batch, we composed a coverage matrix across all samples at 300 bp and 1 kb bin sizes across each chromosome with N-masked bases excluded, then applied cn.MOPS, split raw calls per sample, segregated calls into deletions (copy number < 2) and duplications (copy number > 2) and merged the 300 bp and 1 kb resolution variant predictions per sample per CNV class using *BEDTools merge*.
2. Aberrant alignment signature collection: We collected discordant PE and SR evidence through *svtk collect-pesr*, RD evidence through *svtk bincov* with N-masked regions excluded and BAF evidence from *GATK HaplotypeCaller*-generated VCFs using a custom script (https://github.com/talkowski-lab/CommonMind-SV/blob/master/scripts/vcf2baf.sh). Following evidence collection per sample, we constructed PE, SR, RD, and BAF matrices merged across each phase of sequencing that included 327 (SKL_10073), 326 (SKL_11154), and 119 (SKL_11694) samples, respectively through customized scripts (https://github.com/talkowski-lab/CommonMind-SV/tree/master/Step1b_EvidenceCollection).
3. SV integration and refinement: SV calls detected by each algorithm described above were integrated and calibrated through a series of filtering modules to control the overall FDR and refine variant predictions. Raw outputs from each algorithm were clustered across all samples for each of three sequencing phases (327 samples in SKL_10073; 326 samples in SKL_11154; 119 samples in SKL_11694). Once clustered across samples, the integrated call set was filtered through a random-forest module that tests for statistically significant differences between samples with and without each SV based on four semi-orthogonal signatures: discordant PR and SR reads, RD, and BAF. Finally, filtered, high-quality SV calls were integrated across all three sequencing phases, then we performed alternate allele structure resolution, complex SV classification, other variant refinements, and gene annotation. The filtering module is adaptable to multiple input algorithms, and this same pipeline has been applied to WGS data in ASD families^29^ and population variation datasets^9^.
4. Benchmarking SV accuracy and validation: We have previously benchmarked these SV discovery methods in the gnomAD-SV study^9^, and an earlier iteration of the software was used in a study of autism quartet families^29^. From analyses of 970 trios, we observed a low rate of Mendelian violations (4.2%), consistent with our estimates of <5% FDR from these methods. We further compared our SVs to those generated from long-read genome-sequencing for four samples and observed a 94.0% confirmation rate for 19,316 SVs. Moreover, in our prior study of autism quartets, we performed extensive molecular validation on all de novo SVs predicted in that study, revealing a 97% molecular validation rate for predicted variants in those analyses, which compares well with benchmarking performed in gnomAD-SV.

### SV dataset description

We successfully applied all SV discovery algorithms on 772/773 (99.9%) of the CMC samples, with one failed sample (MSSM-DNA-PFC-375). All 772 samples were included in the SV integration pipeline with SVs assigned in the final call set. The final analyses yielded 125,260 SVs, including 62,948 deletions, 30,547 duplications, 31,155 insertions, 268 simple inversions, 341 complex SVs, and 1 reciprocal translocation. On average, 6220 SVs were identified per sample, consisting of 3579 deletions, 755 duplications, 1839 insertions, 15 inversions, and 14 complex SVs. The number of SVs by class are comparable to other recent SV datasets from short-read genome sequencing^2,9,17,37^. For the insertions, 1146 were further classified as mobile elements insertions, including 1005 Alu, 92 LINE1 and 49 SVA variants. The number of SVs distributed proportionately by read depth among the phases and matched expected demographic history (e.g., 1421 more SVs were detected on average for African-American individuals than all other populations).

### Identification and removal of SV outliers

We carefully examined the set of 772 individuals with SV calls for technical outliers (e.g., related to genome-sequencing generation or biological processes associated with DNA extraction of the postmortem samples). We removed one individual for presence of an abnormal sex chromosome (XXY) which had been previously noted^28^. We further identified 16 samples that represented CNV outliers resulting from anomalous read dosage as calculated by our dosage scoring metric^9^ or if they carried too many or too few CNVs, defined by 3*IQR (interquartile range) of CNVs per individual or genomic content that they alter. After outlier exclusion, we retained 755 (97.8%) of samples with SV data.

### RNA-sequencing pipeline

Samples were processed separately by cohort: CMC and CMC_HBCC^38^.

### RNA-sequencing Re-alignment

RNA-sequencing reads were aligned to GRCh37 with STAR^39^ (v2.4.0g1) from the original FASTQ files. Uniquely mapping reads overlapping genes were counted with featureCounts^40^ (v1.5.2) using annotations from Ensembl v75.

### RNA-sequencing Normalization

To account for differences between samples, studies, experimental batch effects and unwanted RNA-sequencing specific technical variations, we performed library normalization and covariate adjustments using fixed/mixed effects modeling. The workflow consisted of following steps:

1. Gene filtering: Out of ~56 K aligned and quantified genes only those showing at least modest expression were used in this analysis. Genes that were expressed more than 1 CPM in at least 50% of samples in each study were retained for analysis. Additionally, genes with available gene length and percentage GC content from BioMart December 2016 archive were subselected from the above list. This resulted in approximately 14–16 K genes in each batch.
2. Calculation of normalized expression values: Sequencing reads were then normalized in two steps. First, conditional quantile normalization^41^ was applied to account for variations in gene length and GC content. In the second step, the confidence of sampling abundance was estimated using a weighted linear model using voom-limma package in bioconductor^42,43^. The normalized observed read counts, along with the corresponding weights, were used in the following steps.
3. Outlier detection: Based on normalized log2(CPM) of expression values, outlier samples were detected using principal component analysis^44,45^ and hierarchical clustering. Samples identified as outliers using both the above methods were removed from further analysis.
4. Covariate identification: Normalized log2(CPM) counts were then explored to determine which known covariates (both biological and technical) should be adjusted. For the CMC study, we used a stepwise (weighted) fixed/mixed effect regression modeling approach to select the relevant covariates having a significant association with gene expression. Here, covariates were sequentially added to the model if they were significantly associated with any of the top principal components, explaining more than 1% of variance of expression residuals. For CMC_HBCC, we used a model selection based on Bayesian information criteria (BIC) to identify the covariates that improve the model in a greater number of genes than making it worse. That is, covariates were added as fixed effects iteratively in three phases if model improvement by BIC was observed in the majority of genes. Clinical, ancestry and sample-specific technical variables were tested for model improvement first. Covariates related to batch effects were next tested for model improvement and finally, RNA-seq alignment-specific covariates.
5. Surrogate variable analysis adjustments: After identifying the relevant known confounders, hidden-confounders were identified using surrogate variable analysis^46^. We used a similar approach^28^ to find the number of surrogate variables, which is more conservative than the default method provided by the surrogate variable analysis package in R^47^. The basic idea of this approach is to select surrogate variables for removal that explain more variance than expected based on permuted data where all correlation should be random. Thus, from a series of 100 permutations of residuals (white noise) we identified the number of covariates to include. We applied the IRW (iterative re-weighting) version of surrogate variable analysis to the normalized gene expression matrix, along with the covariate model described above to obtain residual gene expression.
6. Covariate adjustments: We performed a variant of fixed/mixed effect linear regression, choosing mixed-effect models when multiple samples, were available per individual, as shown here: gene expression ~ diagnosis + sex + covariates + (1|Donor), where each gene in linearly regressed independently on diagnosis, identified covariates and donor (individual) information as random effect. Observation weights (if any) were calculated using the voom-limma^42,43^ pipeline, which has a net effect of up-weighting observations with inferred higher precision in the linear model fitting process to adjust for the mean–variance relationship in RNA-sequencing data. The Diagnosis component was then added back to the residuals to generate covariate-adjusted expression.

All these workflows were applied separately for each cohort. For CMC_HBCC, samples with age < 18 were excluded prior to analysis.

### Ensuring sample consistency between genome-sequencing and RNA-sequencing

To infer effects on expression from SVs, we had to ensure the genome-sequencing and RNA-sequencing data were from the same individual. To do so we used variant calling data from both platforms. We removed SNVs with missing rate ≥ 0.05 and restricted only to biallelic variants. Upon merging the genotypes from genome-sequencing and RNA-sequencing we calculated genome-wide relatedness from estimates of identity-by-descent using Plink^48^ across all cross-platform pairs of samples. For each genome-sequencing sample we identified the appropriate matching RNA-sequencing sample requiring both near complete relatedness (Pihat > 0.8) and no other sample with high relatedness. Across both cohorts 622/632 (98%) samples matched the expected pair and 10 samples had to be corrected.

### Genomic annotation sources

All data were downloaded in the GRCh37/hg19 build of the human genome. We used TSS definitions from Ensembl v75. To map regions of open chromatin, we used a set of DNase hypersensitive sites downloaded from Roadmap Epigenomics^49^. We mapped the three-dimensional chromatin architecture using TAD domains identified by PsychENCODE from Hi–C contact matrices with 40 kb resolution in the prefrontal cortex (PFC, *n* = 2735)^24^. As a proxy for TAD boundaries or other insulated regions, we used a set of CTCF binding sites from ChIP-seq data downloaded from ENCODE in brain-relevant cell types^50^. We merged overlapping CTCF peaks from each tissue into a single consensus region (*n* = 100,894).

### Comparison of allele frequencies across SV annotation classes

In order to assess whether SVs affecting particular functional elements (genes, enhancers, etc.) were present at lower frequencies than nonfunctional SVs we needed to account for differences in lengths. That is, since longer SVs are more likely to affect functional elements and are rarer (Supplementary Fig. 1) we want to break the dependence on length to assess the role of the functional elements on AF. For each class of SVs affecting a particular annotation we required a matching intergenic SV of the same class that affected none of the annotations with a length within 500 base pairs or 10% of the length of the functional SV, whichever was shorter. We then tested for differences in AF distributions between the two equal numbers of functional and intergenic SVs using the non-parametric Wilcoxon test.

### *Cis*-regulatory element annotations

We downloaded PFC enhancer annotations (*n* = 79,056) from the PsychENCODE project^24^. These were generated by overlapping cross-tissue DNase-seq and ATAC-seq assay information with H3K27ac ChIP-seq peaks. Regions overlapping H3K4me3 peaks and within 2 kb of a TSS were excluded from the set of putative enhancers. All ChIP-seq, ATAC-seq, and DNase-seq data were filtered to include only high-signal peaks with a z-score greater than 1.64. We also downloaded the high confidence set of enhancer annotations (*n* = 18,212) which, in addition to the criteria above, require high PFC H3K27ac ChIP-seq signal (*z*-score > 1.64) in both the PsychENCODE and Roadmap Epigenomics experiments. We generated a set of promoter annotations by using 2 kb windows upstream from each TSS (*n* = 57,773). We intersected these 2 kb windows with PFC H3K27ac from PsychENCODE and PFC H3K4me3 from Roadmap Epigenomics to create a set of high confidence promoters (*n* = 5736)^24,49^. As in the enhancer definition, the H3K27ac and H3K4me3 ChIP-seq data included only high signal peaks with a *z*-score > 1.64.

### Reporting summary

Further information on research design is available in the Nature Research Reporting Summary linked to this article.

## Supplementary information

Supplementary Information

Description of Additional Supplementary Files

Supplementary Data 1

Supplementary Data 2

Reporting Summary

## Data Availability

RNAseq covariates and WGS variant calls are available from the CommonMind Consortium (CMC) Knowledge Portal together with additional data from the CMC cohorts: http://CommonMind.org. Data in the Portal is either open, where the only requirement is to acknowledge data contributors in publications, or controlled. Controlled data access applications must be placed through the NIMH Repository and Genomics Resources (https://www.nimhgenetics.org/resources/commonmind). RNAseq: CMC_HBCC_DLPFC_ResidualExpression.csv: 10.7303/syn22000731.1. CMC_MSSM-Penn-Pitt_DLPFC_ResidualExpression.csv: 10.7303/syn22000700.1. CMC_Human_rnaSeq_metadata.csv: 10.7303/syn18358379.4. WGS: CMC_MSSM-Penn-Pitt-HBCC.SVs.20180426.vcf.gz: 10.7303/syn21914156.2. CMC_Human_WGS_metadata.csv: 10.7303/syn22005337.2. Samples_passingQC.csv: 10.7303/syn22005423.1. Clinical/Demographic: CMC_Human_clinical_metadata.csv: 10.7303/syn3354385.5.

## Code availability

Code for generating regulatory disruption scores is available at https://github.com/RuderferLab/CNV_FunctionalAnnotation. Custom scripts for aberrant alignment signature collection are available at https://github.com/talkowski-lab/CommonMind-SV.
