# Supplementary material for: Functional annotation of rare structural variation in the human brain
Source: Nat Commun. 2020 Jun 12;11:2990. doi: 10.1038/s41467-020-16736-1 (PMC7293301; doi:10.1038/s41467-020-16736-1)
Supplement: Supplementary file 1 — Supplementary Information [file 41467_2020_16736_MOESM1_ESM.pdf]

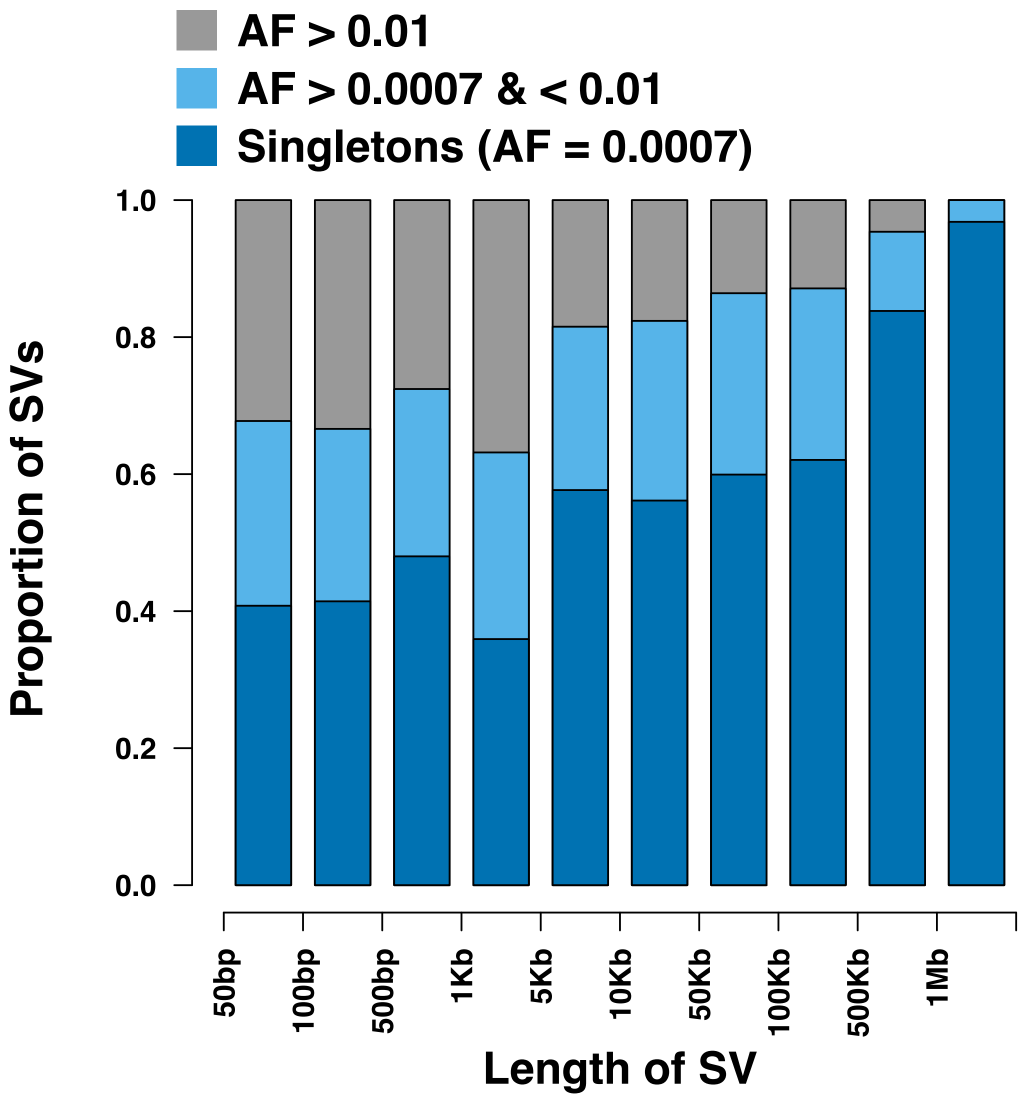

Supplementary Figure 1. Relationship between allele frequency stratified by SV length.

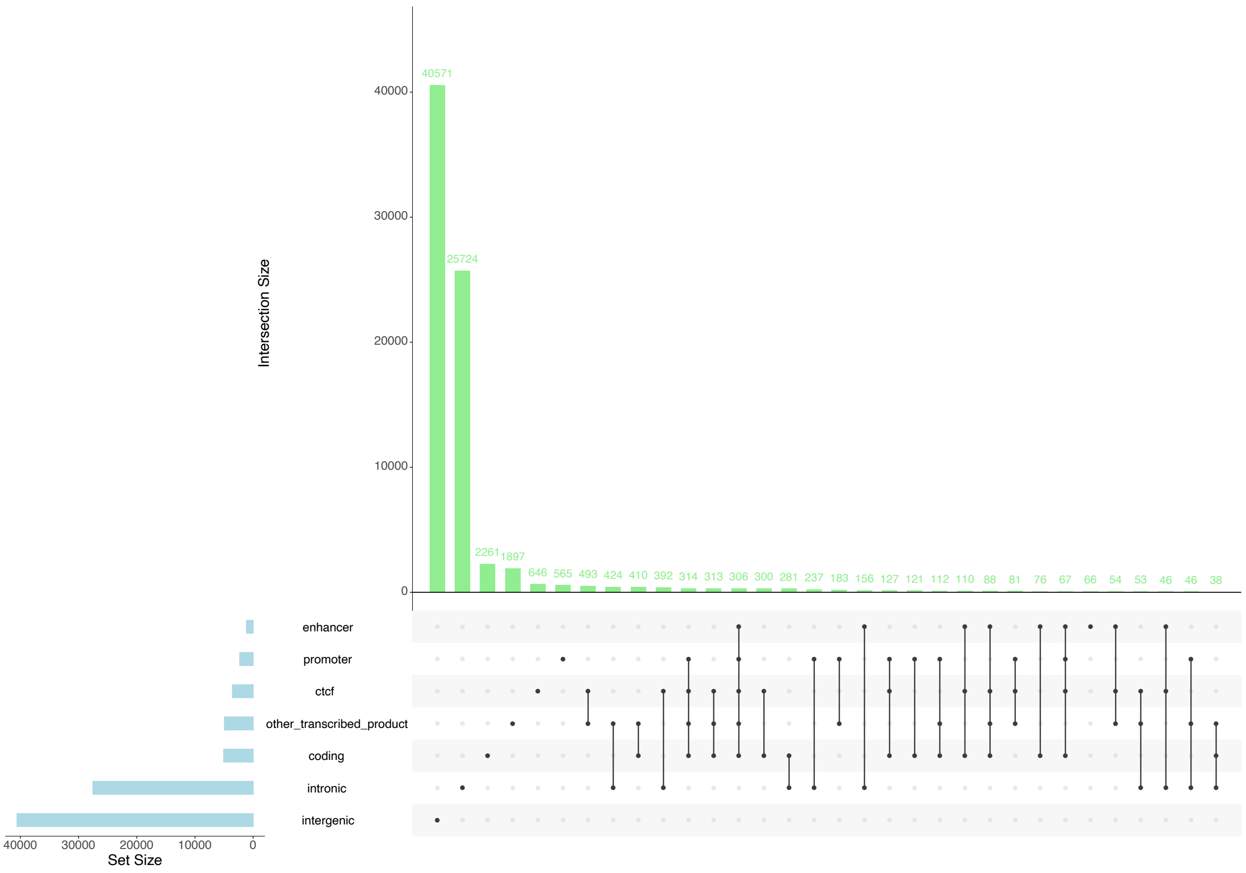

Supplementary Figure 2. Counts of SVs by combination of annotations (green) and full set of any annotation (blue)

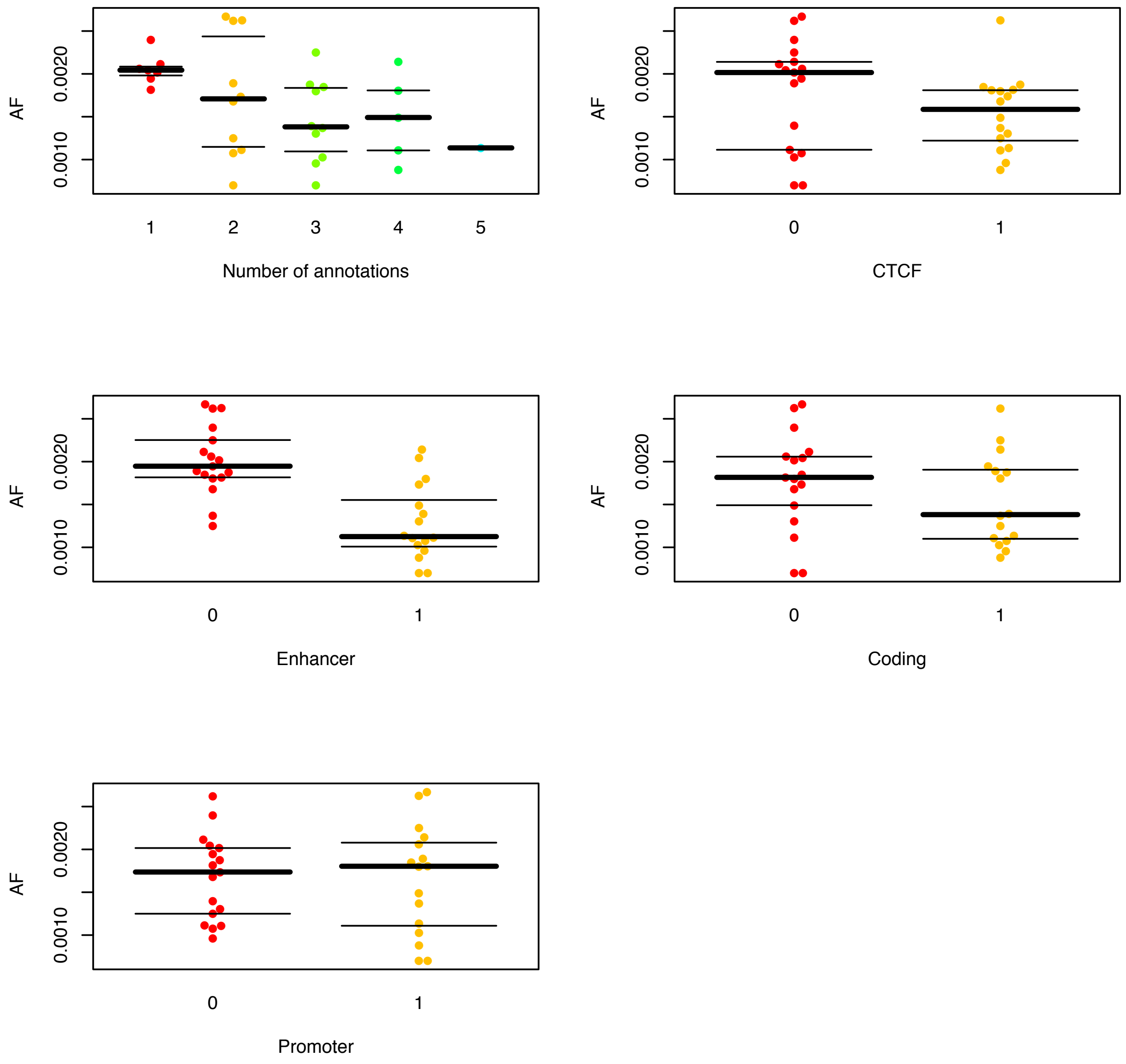

Supplementary Figure 3. Mean allele frequency for each of 33 combinations of annotations split by the number of unique annotations and then by SVs that affect all combinations that include CTCF sites, enhancers, coding genes and promoters. Each point represents an annotation combination (or unique annotation). The thick bar is the median and the thin bars are 95% confidence interval akin to standard boxplot.

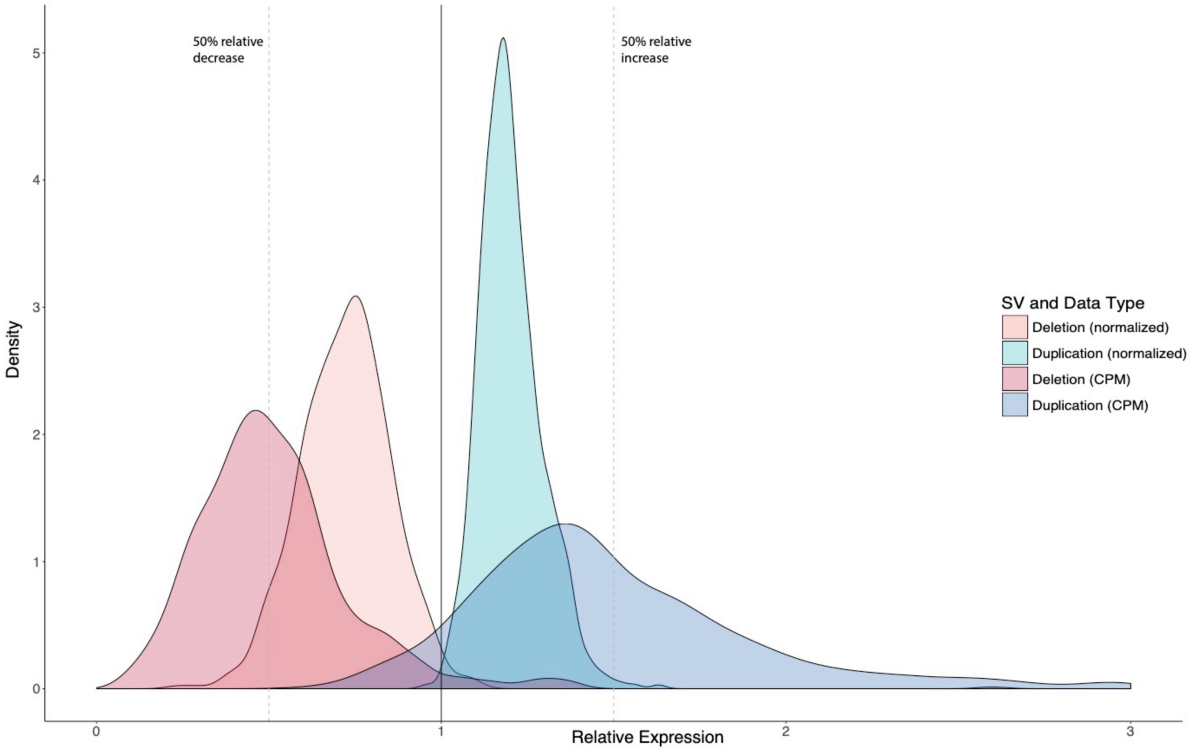

Supplementary Figure 4. Distribution plots of the relative expression of full gene deletions or duplications compared to the rest of the CNV in the normalized and covariate adjusted data (normalized) and in the raw counts per million (CPM). Dashed lines represent expected 50% decrease and 50% increase for full gene deletions and duplications respectively.

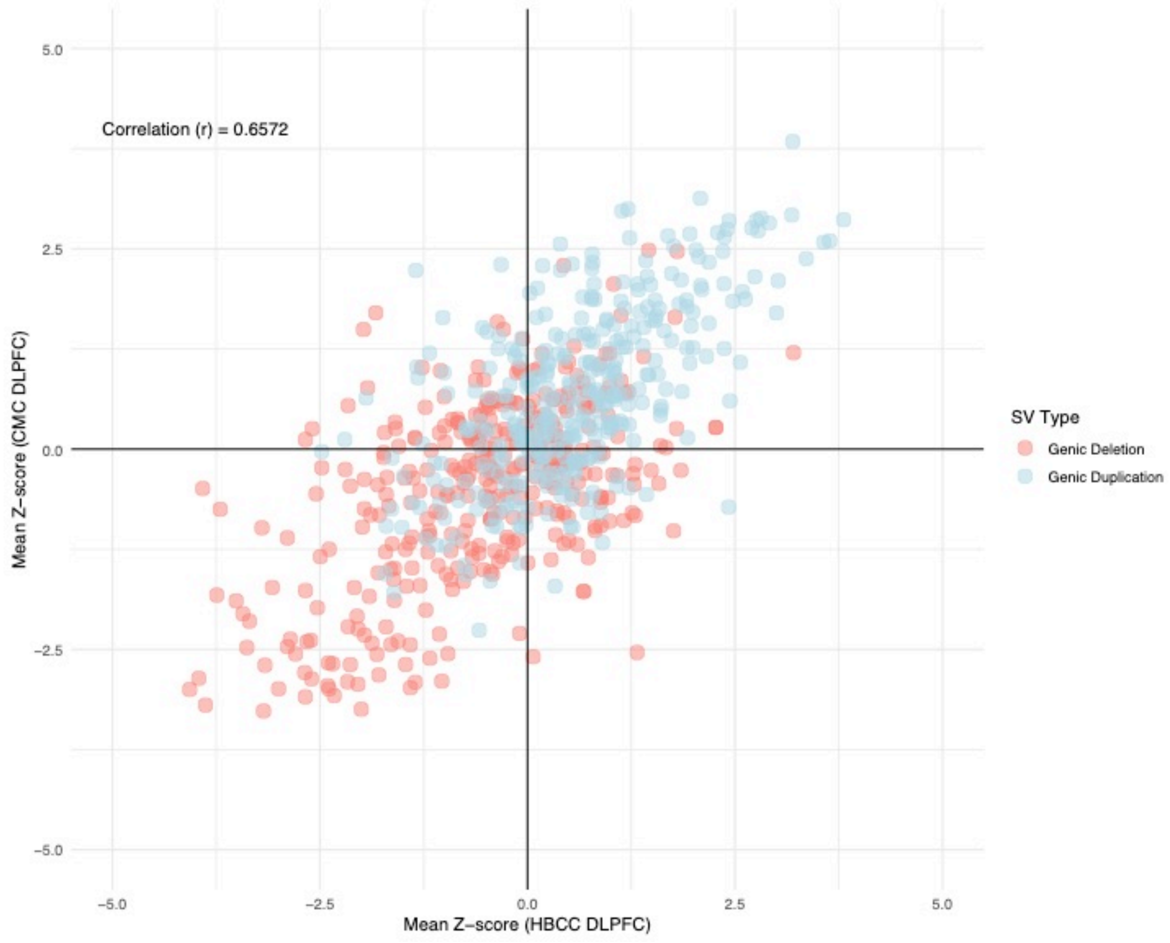

Supplementary Figure 5. Correlation of mean expression values per gene/CNV pair of all CNVs affecting genes between the two independent cohorts (CMC and CMC_HBCC).

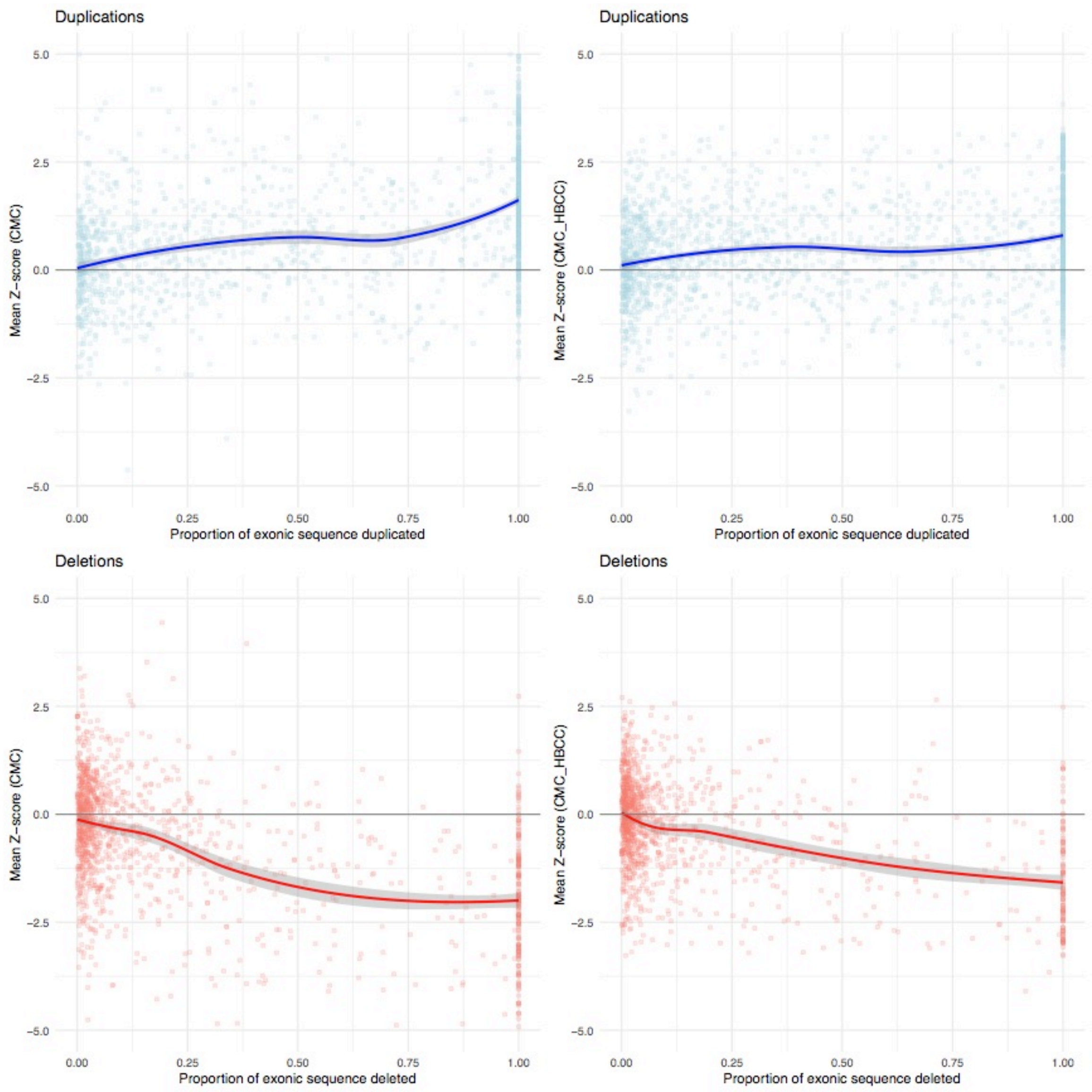

Supplementary Figure 6. Distribution of relative expression (Z-score) as a product of the proportion of exonic sequence of a gene that is deleted (red) or duplication (blue) stratified by cohort. Lines are loess fits and grey area represents the 95% confidence interval of the line.

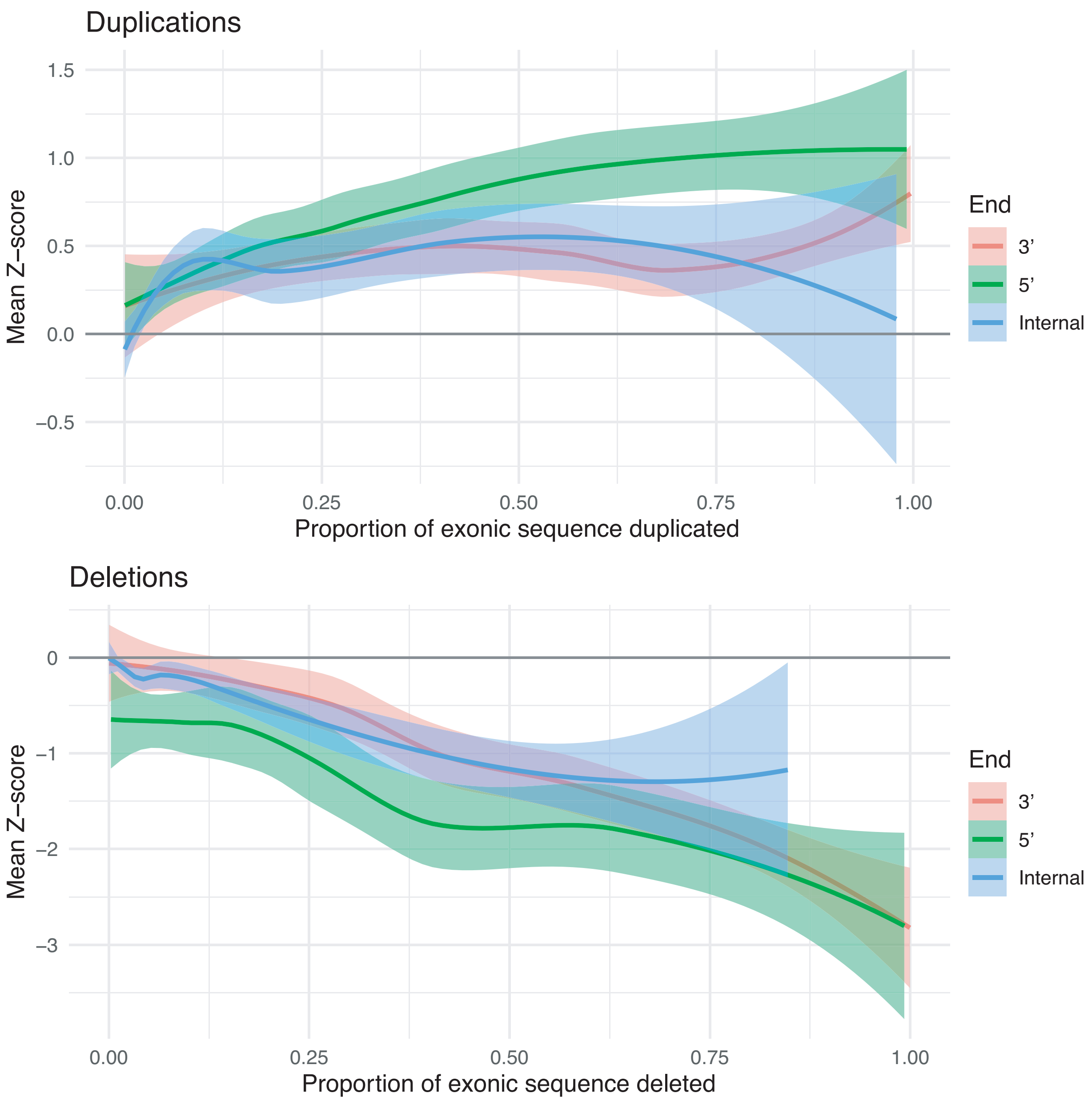

Supplementary Figure 7. Distribution of relative expression (Z-score) as a product of the proportion of exonic sequence of a gene that is deleted or duplication stratified by whether the CNV affected the transcription start (5’), transcription end (3’) or was internal to the exonic sequence. Lines are loess fits and grey area represents the 95% confidence interval of the line.

						Matched to intergenic SV by length						
**SV Type**	**Annot**	**N**	**Length**	**AF**	**Singleton Proportion**	**N**	**Length**	**Length (intergenic)**	**p-value (length)**	**AF**	**AF (intergenic)**	**p-value**
All	coding	5061	83742.3	0.00182	0.582	4618	8884.4	8867.2	0.851	0.00168	0.00193	7.42E-15
	enhancer	1137	267102.2	0.00134	0.706	1055	16584.2	16566.9	0.976	0.00123	0.00196	6.42E-30
	promoter	2297	155880.2	0.00186	0.559	1711	12066.7	12059.4	0.995	0.00193	0.00191	7.00E-01
	ctcf	3544	135901.9	0.00160	0.645	3763	11251.6	11216.5	0.809	0.00161	0.00191	1.00E-16
	intronic	27525	1485.9	0.00201	0.518	29070	1357.5	1363.8	0.086	0.00188	0.00193	4.19E-04
	intergenic	40571	1405.6	0.00211	0.497							
Deletions	coding	2173	37341.5	0.00187	0.586	2296	6498.9	6453.3	0.655	0.00171	0.00184	1.34E-04
	enhancer	421	118339.7	0.00170	0.639	507	10277.2	10236.5	0.819	0.00146	0.00197	6.83E-09
	promoter	873	64859.2	0.00204	0.541	759	6162.4	6164.0	0.998	0.00202	0.00200	6.70E-01
	ctcf	1588	70095.3	0.00178	0.613	2143	8619.5	8555.7	0.710	0.00172	0.00187	1.62E-04
	intronic	19528	1382.6	0.00198	0.530	20489	1298.4	1303.0	0.198	0.00185	0.00189	7.52E-03
	intergenic	17938	1555.3	0.00201	0.517							
Duplications	coding	2439	66000.2	0.00177	0.581	1903	13491.1	13498.2	0.894	0.00163	0.00202	2.46E-12
	enhancer	656	117462.2	0.00110	0.755	529	23180.3	23198.7	0.865	0.00101	0.00199	6.62E-25
	promoter	1145	106414.4	0.00167	0.583	720	21588.8	21589.8	0.991	0.00176	0.00204	8.96E-02
	ctcf	1826	100582.9	0.00144	0.677	1336	17453.0	17425.1	0.936	0.00142	0.00195	3.16E-16
	intronic	5816	1860.1	0.00207	0.498	6198	1882.7	1891.1	0.209	0.00192	0.00197	2.19E-02
	intergenic	5719	3244.4	0.00214	0.476							

Supplementary Table 1. Frequency measures (allele frequency [AF] and singleton proportion within annotation class) of all SVs overlapping a particular annotation, only deletions or only duplications All are then matched 1 to 1 to intergenic SVs based on length and tested for frequency differences using Wilcoxon Rank Sum test.

Annot	N	Length	AF	Singleton Proportion	N	Length	Length (inte	p-value (length)	AF	AF (intergenic)	p-value
coding	2542	4342.8	0.00195	0.552	2874	4673.2	4676.5	0.815	0.00179	0.00197	1.48E-05
enhancer	222	6491.2	0.00204	0.523	260	6109.6	6087.5	0.961	0.00177	0.00192	1.66E-01
promoter	802	675.9	0.00206	0.510	749	704.1	708.0	0.962	0.00207	0.00196	1.29E-01
ctcf	1038	21980.2	0.00182	0.585	2106	8578.1	8562.2	0.914	0.00175	0.00194	1.48E-04
intronic	25724	961.7	0.00202	0.517	27573	923.9	932.0	0.090	0.00189	0.00192	4.34E-03
intergenic	40571	1405.6	0.00211	0.497							

Supplementary Table 2. Frequency measures (allele frequency [AF] and singleton proportion within annotation class) of all SVs overlapping only a single annotation class Both are then matched 1 to 1 to intergenic SVs based on length and tested for frequency differences using Wilcoxon Rank Sum test.

CNV Type	**Variable**	**Est.**	**SE**	**T**	**P**
Deletions	Enhancer sum	-1.41E-02	8.82E-03	-1.597	0.110305
	Promoter proportion	1.98E-01	1.35E-01	1.461	0.143879
	SV Length	-2.23E-07	2.17E-08	-10.26	1.11E-24
	Within TAD	-8.18E-03	2.24E-03	-3.647	0.000266
Duplications	Enhancer sum	-1.08E-02	3.76E-03	-2.862	0.00421
	Promoter proportion	4.94E-01	1.17E-01	4.202	2.64E-05
	SV Length	2.70E-07	2.92E-08	9.214	3.16E-20
	Within TAD	2.71E-03	3.64E-03	0.744	0.45673

Supplementary Table 3. Coefficients of linear model to predict expression z-score after excluding all variants overlapping genes
