# Supplementary material for: Functional annotation of rare structural variation in the human brain
Source: Nat Commun. 2020 Jun 12;11:2990. doi: 10.1038/s41467-020-16736-1 (PMC7293301; doi:10.1038/s41467-020-16736-1)
Supplement: Supplementary file 2 — Description of Additional Supplementary Files [file 41467_2020_16736_MOESM2_ESM.docx]

**Description of Additional Supplementary Files**

**File name:** Supplementary Data 1

**Description:** Top 100 deletions with the most dramatic regulatory disruption score from gnomAD. Includes variables that went into model, maximum proportional overlap with ClinGen variants and ranks by length, number of genes, number of intolerant genes, minor allele frequency and regulatory disruption

**File name:** Supplementary Data 2

**Description:** Top 100 duplications with the most dramatic regulatory disruption score from gnomAD. Includes variables that went into model, maximum proportional overlap with ClinGen variants and ranks by length, number of genes, number of intolerant genes, minor allele frequency and regulatory disruption
