# Supplementary material for: Functional annotation of rare structural variation in the human brain
Source: Nat Commun. 2020 Jun 12;11:2990. doi: 10.1038/s41467-020-16736-1 (PMC7293301; doi:10.1038/s41467-020-16736-1)
Supplement: Supplementary file 5 — Reporting Summary [file 41467_2020_16736_MOESM5_ESM.pdf]

nature research

Corresponding author(s): Douglas Ruderfer

Last updated by author(s): May 5, 2020

Reporting Summary

Nature Research wishes to improve the reproducibility of the work that we publish. This form provides structure for consistency and transparency in reporting. For further information on Nature Research policies, see our Editorial Policies and the Editorial Policy Checklist.

Statistics

For all statistical analyses, confirm that the following items are present in the figure legend, table legend, main text, or Methods section.

n/a	Confirmed	
☐	☒	The exact sample size (*n*) for each experimental group/condition, given as a discrete number and unit of measurement
☐	☒	A statement on whether measurements were taken from distinct samples or whether the same sample was measured repeatedly
☐	☒	The statistical test(s) used AND whether they are one- or two-sided<br>*Only common tests should be described solely by name; describe more complex techniques in the Methods section.*
☐	☒	A description of all covariates tested
☐	☒	A description of any assumptions or corrections, such as tests of normality and adjustment for multiple comparisons
☐	☒	A full description of the statistical parameters including central tendency (e.g. means) or other basic estimates (e.g. regression coefficient) AND variation (e.g. standard deviation) or associated estimates of uncertainty (e.g. confidence intervals)
☐	☒	For null hypothesis testing, the test statistic (e.g. *F*, *t*, *r*) with confidence intervals, effect sizes, degrees of freedom and *P* value noted<br>*Give P values as exact values whenever suitable.*
☒	☐	For Bayesian analysis, information on the choice of priors and Markov chain Monte Carlo settings
☒	☐	For hierarchical and complex designs, identification of the appropriate level for tests and full reporting of outcomes
☐	☒	Estimates of effect sizes (e.g. Cohen's *d*, Pearson's *r*), indicating how they were calculated

Our web collection on statistics for biologists contains articles on many of the points above.

Software and code

Policy information about availability of computer code

Data collection	No software used for data collection
Data analysis	Genome-sequencing: BWA-MEM v0.78, Picard tools v1.83, GATK v3.2.2. SV callling: DELLY (v0.7.5), LUMPY (v0.2.13), Manta (v1.01), Wham (v1.7.0), MELT (v2.1.4), CNVnator28 (v0.3.2), GenomeSTRiP29 (v2), and a custom version of cn.MOPS. RNA-sequencing: STAR v2.4.0g1, featureCounts v1.5.2.

For manuscripts utilizing custom algorithms or software that are central to the research but not yet described in published literature, software must be made available to editors and reviewers. We strongly encourage code deposition in a community repository (e.g. GitHub). See the Nature Research guidelines for submitting code & software for further information.

Data

Policy information about availability of data

All manuscripts must include a data availability statement. This statement should provide the following information, where applicable:

- Accession codes, unique identifiers, or web links for publicly available datasets
- A list of figures that have associated raw data
- A description of any restrictions on data availability

RNAseq covariates and WGS variant calls are available from the CommonMind Consortium (CMC) Knowledge Portal together with additional data from the CMC cohorts: http://CommonMind.org. Data in the Portal is either open, where the only requirement is to acknowledge data contributors in publications, or controlled. Controlled data access applications must be placed through the NIMH Repository and Genomics Resources (https://www.nimhgenetics.org/resources/commonmind).

RNAseq

CMC_HBCC_DLPFC_ResidualExpression.csv: https://doi.org/10.7303/syn22000731.1

• CMC_MSSM-Penn-Pitt_DLPFC_ResidualExpression.csv: https://doi.org/10.7303/syn22000700.1
• CMC_Human_rnaSeq_metadata.csv: https://doi.org/10.7303/syn18358379.4
WGS
• CMC_MSSM-Penn-Pitt-HBCC.SVs.20180426.vcf.gz: https://doi.org/10.7303/syn21914156.2
• CMC_Human_WGS_metadata.csv: https://doi.org/10.7303/syn22005337.2
• Samples_passingQC.csv: https://doi.org/10.7303/syn22005423.1
Clinical/Demographic
• CMC_Human_clinical_metadata.csv: https://doi.org/10.7303/syn3354385.5

Field-specific reporting

Please select the one below that is the best fit for your research. If you are not sure, read the appropriate sections before making your selection.

[x] Life sciences [ ] Behavioural & social sciences [ ] Ecological, evolutionary & environmental sciences

For a reference copy of the document with all sections, see nature.com/documents/nr-reporting-summary-flat.pdf

Life sciences study design

All studies must disclose on these points even when the disclosure is negative.

Sample size	773
Data exclusions	Samples were only excluded if outliers in expression or SV
Replication	Where possible, replication was performed by splitting current sample or using another. Not all analyses could be replicated at present
Randomization	Samples were processed by source and timing. Genome sequencing and RNA-sequencing experiments were performed at different times and in different batches
Blinding	Analysts on this manuscript were not part of batching process

Reporting for specific materials, systems and methods

We require information from authors about some types of materials, experimental systems and methods used in many studies. Here, indicate whether each material, system or method listed is relevant to your study. If you are not sure if a list item applies to your research, read the appropriate section before selecting a response.

Materials & experimental systems

n/a	Involved in the study	
[x]	[ ]	Antibodies
[x]	[ ]	Eukaryotic cell lines
[x]	[ ]	Palaeontology and archaeology
[x]	[ ]	Animals and other organisms
[x]	[ ]	Human research participants
[x]	[ ]	Clinical data
[x]	[ ]	Dual use research of concern

Methods

n/a	Involved in the study	
[x]	[ ]	ChIP-seq
[x]	[ ]	Flow cytometry
[x]	[ ]	MRI-based neuroimaging
